# The Correlation Between Small Dense LDL and Reactive Oxygen Metabolites in a Physical Activity Intervention in Hyperlipidemic Subjects

**DOI:** 10.4021/jocmr870w

**Published:** 2012-05-15

**Authors:** Kazuhiko Kotani, Kokoro Tsuzaki, Naoki Sakane, Nobuyuki Taniguchi

**Affiliations:** a Division of Preventive Medicine, Clinical Research Institute, National Hospital Organization Kyoto Medical Center, Kyoto, Japan; b Department of Clinical Laboratory Medicine, Jichi Medical University, Tochigi, Japan

**Keywords:** Exercise, Dyslipidemia, d-ROMs test, Oxidative stress, LDL particle size

## Abstract

**Background:**

Small dense low-density lipoprotein (sdLDL), which has a small LDL particle size with a greater susceptibility to oxidation, is considered a risk marker for cardiovascular disease (CVD). The diacron reactive oxygen metabolites (d-ROMs) have recently been introduced as a clinically useful oxidative stress-related marker. Physical activity can reduce the CVD risk. The present study investigated the correlation between the changes of the mean LDL particle size and the oxidative stress status, as assessed by the d-ROMs, in a physical activity intervention in hyperlipidemic subjects.

**Methods:**

We performed a 6-month intervention study of 30 hyperlipidemic subjects (12 male/18 female, mean age 64 years), focusing on a moderate physical activity increase. The clinical data, including the atherosclerotic risk factors besides the mean LDL particle size measured with the gel electrophoresis and the d-ROMs, were evaluated pre- and post-intervention.

**Results:**

The mean LDL particle size was significantly larger in the post-intervention than in the pre-intervention evaluation (26.9 ± 0.3 (SD) vs. 27.1 ± 0.4 nm, P < 0.01), while the d-ROMs levels were significantly reduced in the post-intervention period compared to those at pre-intervention (319 ± 77 vs. 290 ± 73 U. Carr., P < 0.05). A stepwise multiple regression analysis revealed that there was an independent, significant and inverse correlation between the pre- and post-intervention changes of the d-ROMs and the mean LDL particle size (β = -0.55, P < 0.01).

**Conclusions:**

The intervention study suggests that sdLDL and oxidative stress can concomitantly affect the risk of developing CVD and that both factors can improve by even a moderate increase in physical activity among hyperlipidemic subjects.

## Introduction

Cardiovascular disease (CVD) remains a major sociomedical burden, and the regulation of incident CVD is a public-health challenge [1]. CVD is a multi-factorial disorder, and hyperlipidemia is also associated with the development of CVD [1]. In addition to the classic atherosclerotic risk factors, lipoprotein disorders, including the existence of small dense low-density lipoprotein (sdLDL), and the presence of oxidative stress are considered to be the risk factors, and have attracted increasing attention in recent years [2-7]. Regular physical activity can reduce the CVD risk, although the biological mechanisms underlying this reduction remain incompletely elucidated [8-10].

Small dense LDL particles (showing a smaller LDL particle size) are highly susceptible to oxidation, and their oxidation concept is related to the atherogenic potential of sdLDL particles and is associated with incident CVD [2, 3, 5, 6]. In general, regular physical activity improves lipoprotein/lipid disorders in adults [11]. Persistent intervention studies specific for regular exercise have reported a significant reduction of sdLDL levels (that is, indicating a change toward a larger LDL particle size) [12, 13], although a cross-sectional study has conflictingly reported a non-significant difference in sdLDL levels between habitual exercisers and sedentary male subjects [14]. However, because there are complex interdependencies between many atherosclerotic risk factors, whether the influence of sdLDL on the development of CVD is independent of other atherosclerotic risk factors is still being debated [6].

Oxidative stress is present when an oxidative environment develops due to an imbalance between oxidative reactants, such as reactive oxygen species (ROS), and antioxidants [15-17]. While oxidative stress is considered to be involved in the development of CVD [4, 7], several studies specific for exercise have reported that regular exercise results in a reduction of malondialdehyde and/or lipid peroxidation products as oxidative stress-related markers in diseased patients [18-21]. However, studies using oxidative stress have had limited indices available to easily analyze the oxidative stress status of patients in the clinical setting [16, 17]. The recently introduced diacron reactive oxygen metabolites (d-ROMs) test (Diacron, Italy) can quantify the oxidative stress status by measuring the levels of hydroperoxides of organic compounds (lipids, proteins, nucleic acids, etc.), and has been used as a simple clinical marker of oxidative stress [22-24]. However, earlier studies on exercise using the d-ROMs test have been only cross-sectionally conducted for players of specific sports [25-27].

In this context, although physical activity can reduce the respective markers of sdLDL and the oxidative stress status, more data are necessary to confirm the importance of physical activity with regard to both of these markers. Little clinical information is available about the cross-linkage between sdLDL and oxidative stress from an intervention study on physical activity. We hypothesize that concomitant changes may be seen in sdLDL and oxidative stress, as assessed by the d-ROMs test, as a result of an increase in physical activity. Because their association is still unproven, the aim of the present study was to investigate the correlation between the mean LDL particle size and the d-ROMs levels among hyperlipidemic subjects during a physical activity intervention period.

## Methods

A 6-month intervention study was performed that included 30 hyperlipidemic patients (12 male and 18 female, mean ages: 64 years). Hyperlipidemia was diagnosed according to the guidelines of the Japan Atherosclerosis Society (circulating concentrations of LDL cholesterol (LDL-C) ≥ 3.64 mmol/L, triglycerides (TG) ≥ 1.69 mmol/L) [28]. The inclusion criteria was non-smokers, patients with regular exercise habits (structured exercise < 30 minutes, twice a week), and patients not taking any medications. The exclusion criteria were individuals who were pregnant, had acute infections such as the common cold, were alcohol abusers, and those who had a history of diabetes, cardio/cerebrovascular, thyroid, collagen, severe kidney or liver diseases. The study was approved by the institutional ethics committee, and all subjects gave their informed consent.

The present program, which focused on a moderate physical activity increase, included monthly group sessions to promote physical activity, such as daily walking, and to instruct subjects on the appropriate methods by exercise specialists. In particular, the participants were educated on how to measure their heart rates during a walking period in their radial arterial pulses, and were recommended to walk at 60% of their maximal heart rate for at least 30 minutes.

In the pre- and post-intervention examinations, atherosclerotic risk variables were measured during an overnight fasted state. In addition to the body mass index (BMI), the systolic blood pressure (SBP) and diastolic blood pressure (DBP) was measured in the right arm of each subject in the seated position after a 5-minute rest using a sphygmomanometer. The serum lipid panels such as the LDL-C, TG and high-density lipoprotein cholesterol (HDL-C) and plasma glucose levels were measured using enzymatic methods. The mean LDL particle size was measured with a high-resolution, nongradient polyacrylamide gel electrophoresis system (the Lipoprint system; Quantimetrix, Redondo Beach, CA, USA), which has been validated by using the gold standard method of nuclear magnetic resonance spectroscopy. Briefly, after serum samples (25 μL) were photopolymerized, the samples and loading gels were applied to gel tubes and then electrophoresed. The scanning system calculated the mean LDL particle size based on the fractionalized lipoproteins [29]. Furthermore, the d-ROMs values were measured using a kinetic spectrophotometric assay (the F.R.E.E system; Diacron, Italy) with intra- and inter-assay coefficients of variation of 2.1% and 3.1%, respectively [22, 23]. Briefly, serum samples (25 μL) were mixed with a buffered solution, and a chromogenic substrate was added to the mixture. The mixture was then incubated in the thermostatic block of the system. The absorbance was recorded at 505 nm. The measurements are expressed in U. Carr., where 1 U. Carr. corresponds to 0.08 mg/dL H_2_O_2_.

The data are expressed as the means ± standard deviations (SD) or the medians plus interquartile ranges. Paired t-tests were used to compare the pre- and post-intervention levels of the respective variables. A Pearson’s correlation test for a simple univariate analysis and a stepwise multiple regression analysis adjusted for all of the listed variables were utilized to observe the correlation between the pre- and post-intervention changes of the d-ROMs levels and other atherosclerotic risk variables, including the mean LDL particle size. The values of TG were log-transformed for all the analyses because of their skewed distribution. A P-value ≤ 0.05 was considered to be statistically significant.

## Results

The clinical characteristics of the studied subjects are listed in Table 1. There was a significant reduction of the BMI, LDL-C and d-ROMs levels, while there was a significant increase of the mean LDL particle size, during the physical activity intervention period.

**Table 1:** Measured Variables at the Pre- and Post-Intervention Examinations of Physical Activity in Hyperlipidemic Subjects

Variable	Pre-intervention	Post-intervention	P-value
Age, years	64 ± 6	-	
Male/female, number	12/18	-	
Body mass index, kg/m^2^	23.5 ± 3.0	23.1 ± 2.7	0.01**
Systolic blood pressure, mmHg	139 ± 19	138 ± 21	0.79
Diastolic blood pressure, mmHg	77 ± 12	79 ± 15	0.32
Fasting plasma glucose, mmol/L	5.47 ± 0.82	5.37 ± 0.73	0.28
LDL-cholesterol, mmol/L	3.83 ± 0.50	3.44 ± 0.62	0.01**
Triglycerides, mmol/L	1.24 (0.99-1.53)	1.20 (0.82-1.41)	0.17
HDL-cholesterol, mmol/L	1.76 ± 0.40	1.78 ± 0.41	0.74
Mean LDL particle size, nm	26.9 ± 0.3	27.1 ± 0.4	0.01**
d-ROMs, U. Curr.	319 ± 77	290 ± 73	0.02**

LDL: low-density lipoprotein, HDL: high-density lipoprotein, d-ROMs: diacron reactive oxygen metabolites. The data are shown as the means ± standard deviation or medians (interquartile range). A paired t-test was used to analyse the respective markers. Significance level: * P ≤ 0.05, ** P ≤ 0.01.

The correlations between the pre- and post-intervention changes in the d-ROMs and atherosclerotic risk variables are listed in Table 2. A simple univariate correlation test showed that the change in the d-ROMs levels was significantly and positively correlated with that of the BMI and TG, while the change in the d-ROMs was significantly and inversely correlated with that of the mean LDL particle size. A subsequent stepwise multiple regression analysis for the change in the d-ROMs levels revealed an independent, significant and inverse correlation between the change of the d-ROMs levels and the mean LDL particle size, although an independent, significant and inverse correlation of age with the change in the d-ROMs was also observed.

**Table 2:** The Correlations of the Changes in the D-ROMs With Other Atherosclerotic Risk Variables, Including the Mean LDL Particle Size, in Hyperlipidemic Subjects

Variables	γ (P-value)	β (P-value)
Age, years	-0.17 (0.38)	-0.34 (0.05)*
Gender, male	0.05 (0.81)	Not extracted
Δ Body mass index, kg/m^2^	0.40 (0.03)*	Not extracted
Δ Systolic blood pressure, mmHg	-0.34 (0.07)	Not extracted
Δ Diastolic blood pressure, mmHg	-0.17 (0.37)	Not extracted
Δ Fasting plasma glucose, mmol/L	0.13 (0.50)	Not extracted
Δ LDL-cholesterol, mmol/L	0.24 (0.21)	Not extracted
Δ Tyiglecerides, mmol/L	0.38 (0.04)*	Not extracted
Δ HDL-cholesterol, mmol/L	-0.25 (0.19)	Not extracted
Δ mean LDL particle size, nm	-0.44 (0.02)*	-0.55(0.01)**

d-ROMs: diacron reactive oxygen metabolites, LDL: low-density lipoprotein, HDL: high-density lipoprotein; r: simple correlation coefficient between the changes of the d-ROMs and another variable, β: stepwise multiple regression coefficient of the change of the d-ROMs with another variable after adjusting for all the listed variables. The triglycerides values were log-transformed for the analyses. Significance level: *P ≤ 0.05, **P ≤ 0.01.

## Discussion

The present study showed that there was an independent, significant and inverse correlation between the changes in the mean LDL particle size and the oxidative stress status, as evaluated by the d-ROMs test, during the physical activity intervention period in hyperlipidemic subjects. The present physical activity produced a significant increase in the mean LDL particle size and a significant reduction of the d-ROMs levels. While this was expected based on the findings of previous studies [12, 13, 18-21], these findings support the beneficial influence of physical activity on lipoprotein metabolism and the oxidative stress status. On the other hand, the precise biological influence of regular physical activity on the CVD risk reduction remains to be determined [8-10], and the sdLDL and oxidative stress-related markers are associated with some issues that need to be resolved (i.e., independency of the sdLDL-CVD association with other atherosclerotic risk factors [6] and the need for simple and useful markers to evaluate the oxidative stress status in the clinical setting [16, 17]). In addition, there have been few clinical intervention studies on physical activity available performed to examine the cross-talk between sdLDL and oxidative stress. Therefore, it is valuable to note that our intervention study demonstrates that concomitant changes of sdLDL and oxidative stress can be associated with the reduction of the development of CVD, even when there is only a moderate increase in physical activity among hyperlipidemic subjects.

Small dense LDL can be formed in the *in vivo* environment due to an increase in the oxidative stress status, that resulting from a sedentary lifestyle [6, 24]. An increase in physical activity might thus decrease the oxidative environment. In addition, sdLDL *per se* can enhance oxidative stress [2, 3, 6]. Because of their low affinity for the LDL receptor, their prolonged half-life in the circulation and their low resistance to oxidative stress, sdLDL particles are more easily taken up in the arterial walls and display a high susceptibility to oxidation with their retention in the walls, leading to uptake by macrophages, and thereafter, foam cell formation [2, 3, 6]. The atherosclerotic processes produce oxidative stress [30]. Physical activity might accelerate the clearance of circulating sdLDL particles via increased lipase activities and attenuate the atherosclerotic processes in the arterial walls [11, 31]. More research is needed to clarify the biological mechanisms responsible for the correlation between sdLDL and oxidative stress.

There are some limitations associated with this study. Even though this was a prospective intervention study, a randomized-controlled design was not employed and there were no control subjects. The number of subjects was relatively small, and the intervention period was relatively short. The intensity and amount of physical activity were not fully evaluated in this study. Therefore, these limitations will need to be addressed in future studies.

In summary, the present intervention study showed that there was an independent, significant and inverse correlation between the changes in the mean LDL particle size and the d-ROMs levels during a 6-month period of increased physical activity in hyperlipidemic subjects. The finding of a correlation between the changes in both markers suggests that sdLDL and oxidative stress can concomitantly affect the reduction of the CVD risk by even a moderate increase in physical activity in hyperlipidemic subjects. Further studies are required to confirm the observed relationship.
